# Sarcomatoid-associated gene risk index for clear cell renal cell carcinoma

**DOI:** 10.3389/fgene.2022.985641

**Published:** 2022-09-08

**Authors:** You Zuo, Shuai Fu, Zhongwei Zhao, Zeyan Li, Yijian Wu, Tienan Qi, Jianguo Zheng, Qinglong Du, Zhonghua Xu, Nengwang Yu

**Affiliations:** ^1^ Department of Urology, Qilu Hospital of Shandong University, Jinan, Shandong, China; ^2^ Cheeloo College of Medicine, Shandong University, Jinan, Shandong, China; ^3^ Department of Medical Oncology, Shandong Cancer Hospital and Institute, Shandong First Medical University and Shandong Academy of Medical Sciences, Jinan, China

**Keywords:** TCGA, sarcomatoid renal cell carcinoma, clear cell carcinoma, nomogram, prognosis

## Abstract

Sarcomatoid renal cell carcinoma is a de-differentiated form of kidney cancer with an extremely poor prognosis. Genes associated with sarcomatoid differentiation may be closely related to the prognosis of renal cell carcinoma. The prognosis of renal cell carcinoma itself is extremely variable, and a new prognostic model is needed to stratify patients and guide treatment. Data on clear cell renal cell carcinoma with or without sarcomatoid differentiation were obtained from TCGA database, and a sarcomatoid-associated gene risk index (SAGRI) and column line graphs were constructed using sarcomatoid-associated genes. The predictive power of the SAGRI and column line graphs was validated using an internal validation set and an independent validation set (E-MTAB-1980). The SAGRI was constructed using four sarcoma-like differentiation-related genes, COL7A1, LCTL, NPR3, ZFHX4, and had a 1-year AUC value of 0.725 in the training set, 0.712 in the internal validation set, and 0.770 in the independent validation set for TCGA training cohort, with high model reliability. The molecular characteristics among the SAGRI subgroups were analyzed by multiple methods, and results suggested that the SAGRI-HIGH subgroup may benefit more from immunotherapy to improve prognosis. SAGRI satisfactorily predicted the prognosis of patients with clear cell renal cell carcinoma with or without sarcomatoid differentiation.

## Introduction

Kidney cancer accounts for approximately 4% of all human malignancies. Approximately 180,000 people die from kidney cancer worldwide each year, and this number is increasing (Bray et al., 2018). The degree of malignancy of kidney cancer itself varies widely, and a significant number of small kidney cancers are not detected owing to their inert behavior (Wunderlich et al., 1998). However, up to 30% of patients can develop recurrence or metastasis after surgery, significantly affecting prognosis (Lam et al., 2005; Lucca et al., 2015; Haas et al., 2016)

Therefore, it is imperative to construct a more accurate risk stratification system for kidney cancer to guide treatment strategies. At present, the common international kidney cancer staging method is the TNM stage system of the American Joint Committee on Cancer (AJCC). This staging incorporates tumor size and local invasion (T), lymph node metastasis (N), and distant metastasis (M), which only approximately reflects the cross-sectional invasion status at the time of kidney cancer diagnosis and cannot longitudinally reflect biological malignancy. The system has been continuously updated and refined as its limitations have been identified in practice; it is now in the eighth edition since its introduction. Another widely used risk stratification system for limited kidney cancer was developed by the University of California, Los Angeles (University of California, Los Angeles Integrated Staging System (UISS)), which incorporates additional physical scores and pathological grading of kidney cancer compared to the AJCC system. However, it still does not accurately reflect the heterogeneity of kidney cancer. The scoring system constructed by the Mayo Clinic for the prediction of outcomes after radical resection for renal clear cell carcinoma incorporates renal cancer stage, size, grade, and necrosis (Frank et al., 2002), but the C-index in external validation ranges from 0.6 to 0.8 (Parker et al., 2017), and there remains a great need for improvement (Mischinger et al., 2019). For limited renal clear cell carcinoma, the ClearCode34 system was recently developed based on the grouping of 34 genes with different expression profiles (Ghatalia and Rathmell, 2018). This system is less discriminatory than the traditional UISS (C-index 0.62 vs. 0.83) (Zigeuner et al., 2010). Therefore, new models for the risk stratification of kidney cancer are needed.

Approximately 90% of all diagnosed renal parenchymal malignancies are renal cell carcinoma (RCC), which includes subtypes such as clear cell carcinoma, papillary carcinoma, and suspicious cell carcinoma (Fang et al., 2019; Li et al., 2021). A rare transformation called sarcomatoid dedifferentiation can occur in most RCC histologic subtypes and portends a particularly poor prognosis. RCC that undergoes sarcomatoid dedifferentiation is often referred to as sarcomatoid renal cell carcinoma (sRCC). Patients with sRCC are often diagnosed in the advanced stage or have metastases, and rarely survive more than 1 year (Delahunt, 1999; Adibi et al., 2015). Approximately 20% of patients with metastatic renal cell carcinoma have concomitant sarcomatous differentiation (Shuch et al., 2009). sRCC has a very poor prognosis, with approximately 60–80% of patients diagnosed in the advanced stage or inoperable (Shuch et al., 2009; Alevizakos et al., 2019). The median survival is approximately 6–13 months, and the higher the percentage of sarcomatoid differentiation, the worse the patient’s prognosis (Shuch et al., 2009; Alevizakos et al., 2019). Regardless of stage, patients with sRCC have a worse survival rate than kidney cancer patients without sarcomatoid differentiation (Golshayan et al., 2009). The subtype in which sarcomatoid differentiation occurs is closely related to the traditional histologic subtype of kidney cancer (Delahunt et al., 2013; Moch et al., 2016). Therefore, sarcomatoid differentiation is now considered to be a “dedifferentiation” pattern, a change from the traditional histologic subtype of kidney cancer that has lost its epithelial features (Delahunt, 1999). Because of the high overall prevalence of clear cell renal cell carcinoma (ccRCC), the majority of sRCCs detected are transformed from the ccRCC subtype (Shuch et al., 2012; Klatte et al., 2018). Sarcomatoid differentiation is closely associated with poor prognosis in renal cell carcinoma. Therefore, we constructed a prognostic model of clear cell renal cell carcinoma by identifying genes associated with sarcomatoid differentiation.

## Materials and methods

### Data acquisition and preprocessing

The case data for this study were obtained from The Cancer Genome Atlas (TCGA) database, and TCGA data were downloaded from the “TCGAbiolinks” package in R language. External validation of the model was done using the E-MTAB-1980 cohort (www.ebi.ac.UK) (Sato et al., 2013). Because the open-access data do not include patient-identifiable information, ethical clearance was not required. Matching transcriptomic data with clinical data were conducted, and missing data of either type, as well as duplicate samples, were removed.

### Identification of genes associated with sarcoma-like differentiation

X-tile (Camp et al., 2004) converts continuous variables into ordered categorical variables by taking the best truncation values for continuous variables based on survival outcomes.

The propensity score matching (PSM) analysis in this study was implemented using the “MatchIt” package (version 4.3.2) in R. The settings were logistic regression for score calculation, best proximity method for matching, and a caliper value of 0.02. Only successfully matched samples will be included in the transcriptome difference analysis. Variables that differed between the sarcomatoid renal cell carcinoma and clear cell renal cell carcinoma groups were included in the calculation.

For paired cases of sRCC and ccRCC after propensity score matching, mRNA differential expression was compared between the two groups. The analysis was performed using the R language software package “edgeR”. The screening result conditions were set as |logFC| ≥ 2 and FDR ≤0.05 (Zhang et al., 2020a; Zhang et al., 2020b).

### Constcruction of the sarcomatoid-associated gene risk index model

The differentially expressed mRNAs between the sRCC and ccRCC groups were labeled sarcomatoid associated genes, and all TCGA samples were randomly divided into training and validation sets at a ratio of seven to 3. The model was constructed using TCGA training set, and one-way Cox regression was used to analyze the genes associated with sarcomatoid differentiation and those associated with prognosis, with a *p*-value of less than 0.05 used as the screening criteria. Lasso regression analysis was then performed, and the prognosis-related genes obtained in the first step were further downscaled. Then, the genes obtained from Lasso regression were subjected to multi-factor COX regression analysis to construct the model for screening factors associated with independent prognostic effects. The sarcomatoid-associated gene risk index (SAGRI) was calculated based on the regression coefficients, and then divided into high risk and low risk groups based on the cut-off values. The effects of clinical information and risk models on prognosis were examined using one-way COX regression and multi-way Cox regression, respectively. Factors that had an impact on prognosis were included in the construction of the nomogram, and ROC curves were plotted along with other clinical information. The risk model and nomogram were validated using TCGA internal validation set and the E-MTAB-1980 external validation set.

### Immune checkpoints

After reviewing the literature, 49 immune checkpoint-related genes were identified (Table), and statistical analysis was performed using the R language “limma” package with a non-parametric test (Wilcoxon test) to compare the expression of immune checkpoint genes in the sRCC and ccRCC groups. TIDE scores were calculated using an online website (http://tide.dfci.harvard.edu/) to estimate the effect of the sample on immunotherapy.

### Analysis of immune cell content and immune function

In this study, estimation of immune cell content and immune function of the samples was performed using single-sample gene set enrichment analysis (ssGSEA). ssGSEA is an extension of the GSEA method, which is free from the limitation that enrichment analysis cannot be performed on a single sample. ssGSEA allows for the estimation of ssGSEA and can score samples for relevant function or expression based on the available gene set. The enrichment analysis was performed using the “GSVA” (version 1.40.1) and “GSEABase” (version 1.54.0) packages in R.

### Identification of SAGRI hub genes

Differential genes were analyzed between SAGRI-HIGH and SAGRI-Low using the R package “limma”, and the screening conditions were |logFC| ≥ 2 and FDR ≤0.05. The differential genes obtained were subjected to GO and KEGG enrichment analysis. Correlations between genes were analyzed using the online website (https://cn.string-db.org/), and the minimum required interaction score was set to high confidence (0.7). The hub genes were identified using Cytoscape software. Survival curves of the hub genes in TCGA cohort were then plotted.

## Results

### TCGA cohort data processing

The clinical information of the 537 KIRC patients were downloaded from TCGA database using the R language “TCGAbiolinks” package, and duplicate data and missing data were removed. Data from a total of 454 were ultimately obtained. The original pathology reports of patients in TCGA database were obtained from the Digital Slide Archive website (http://cancer.digitalslidearchive.net/), and the pathology reports were read manually to select cases with sarcomatoid differentiation. A total of 45 cases of renal clear cell carcinoma with sarcomatoid differentiation (sRCC) and 409 cases of renal clear cell carcinoma without sarcomatoid differentiation (ccRCC) were also included in the study in conjunction with a study on sRCC in TCGA (Bakouny et al., 2021).

**FIGURE 1:**
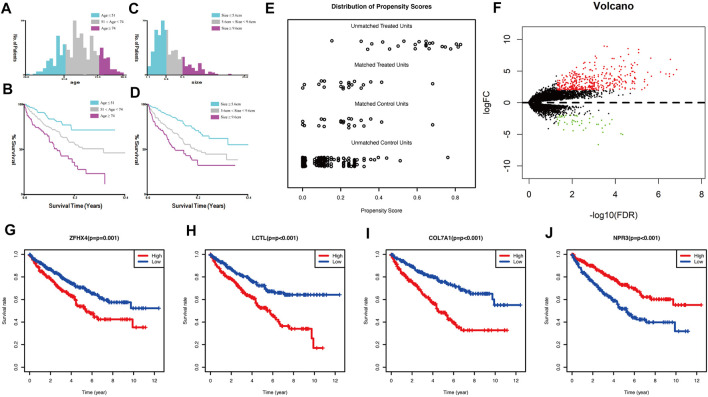
**(A)** Classification result of age using x-tile; **(B)** Kaplan-Meier survival curve of age classification; **(C)** classification result of size using x-tile; **(D)** Kaplan-Meier survival curve of size classification; **(E)** propensity score matching result of two groups of sRCC and ccRCC; **(F)** volcano plot of mRNA expression differences between sRCC and ccRCC; **(G–J)**: Kaplan-Meier survival curve of the four genes in TCGA cohort used for constructing the model.

Continuous variables such as age and tumor size were transformed into categorical variables by taking cut-off values using X-tile for such continuous variables. The results are shown in Figures 1A–D. Age was divided into three groups: less than or equal to 51 years, 52–73 years, and greater than or equal to 74 years; tumor size was divided into three groups: less than or equal to 5.6 cm, 5.7–9.5 cm, and greater than or equal to 9.6 cm.

### Identification of sarcoma-like related genes

The clinical data of unmatched sRCC and ccRCC patients were first compared, and the Chi-square test or exact test was selected according to the frequency; results are shown in Supplementary Table S1. Statistical differences were found in Stage, T stage, N stage, M stage, and tumor size between the two groups. A total of 21 cases of sRCC and 21 cases of ccRCC were successfully matched. Pre-post comparisons of the matching PSM scores are shown in Figure 1E. Results showed that the balance of baseline information between sRCC and ccRCC was improved significantly after matching.

The differences between the sRCC and ccRCC groups after PSM matching were analyzed using the “edgeR” package in R. This package was used to perform differential analysis of genes after removing under-expressed genes and performing TMM (trimmed mean of M values) normalization correction. The results were plotted as volcano plots using the R package The “gplots”, are shown in Figure 1F. Ultimately, 393 genes were upregulated, and 46 genes were downregulated in sRCC. These 439 genes were defined as sarcoma-like differentiation-related genes.

### Construction of the SAGRI and nomogram graphs

A prognostic stratification model for kidney cancer was constructed using sarcoma-like differentiation-associated genes. The 454 TCGA-KIRC samples were randomly divided into a training set (N = 319) and a validation set (N = 135) at a ratio of 7: 3. Models were constructed using TCGA training set cohort.

**FIGURE 2:**
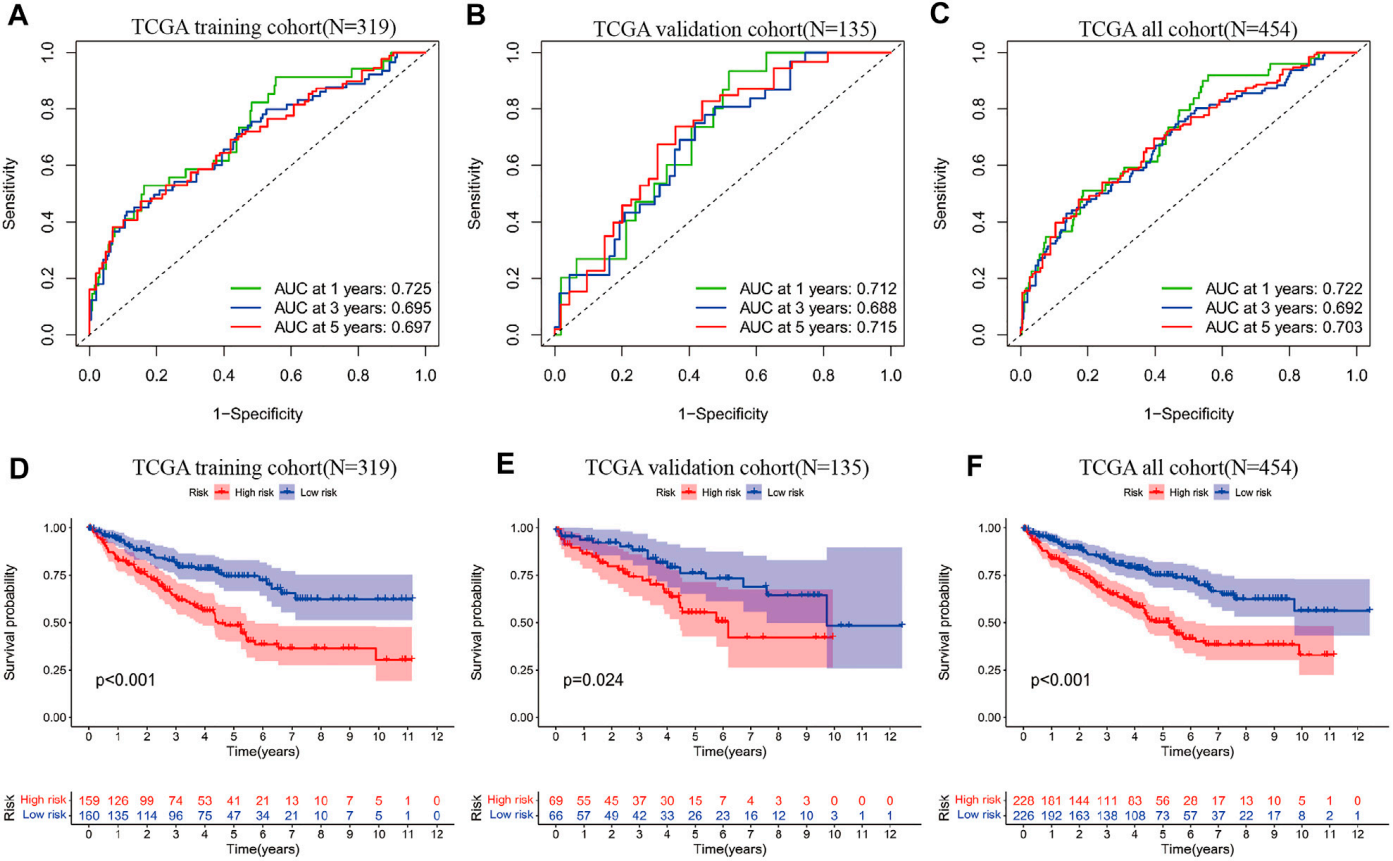
**(A)** ROC curves of SAGRI in TCGA training cohort; **(B)** ROC curves of SAGRI in TCGA validation cohort; **(C)** ROC curves of SAGRI in the full TCGA cohort; **(D)** K-M survival curves of SAGRI-HIGH and SAGRI-LOW in TCGA training cohort; **(E)** K-M survival curves of SAGRI-HIGH and SAGRI-LOW in TCGA validation cohort; **(F)** K-M survival curves of SAGRI-HIGH and SAGRI-LOW in the full TCGA cohort.

Genes associated with prognosis were first screened using one-way COX regression analysis, and 183 genes were screened from the 439 genes associated with sarcoma-like differentiation and prognosis. Then, Lasso regression was used to further downscale the number of genes to obtain four genes, COL7A1, LCTL, NPR3, and ZFHX4. Plotting the survival curves of these four genes separately also showed that the high expression of COL7A1, LCTL, and ZFHX4 predicted poor prognosis, while high expression of NPR3 represented a better prognosis; the difference between high and low expression was obvious (Figures 1G–J). A stratified prognostic model was then constructed using multifactorial Cox regression with the SAGRI calculation formula SAGRI = COL7A1 × 0.0512269863,293,571 + LCTL × 2.31801428021911 + NPR3 × (−0.0167625372671674) + ZFHX4 × 0.295409105635163. The median SAGRI value was taken as the cut-off point and the training set patients were divided into two groups with high and low risk. The risk formula and cutoff values were obtained using TCGA training set to calculate the stratification of TCGA internal validation set and the E-MTAB-1980 external validation set. The survival curves and ROC curves for TCGA training set, TCGA validation set, and TCGA overall were plotted as shown in Figure 2. The area under the curve (AUC) values for TCGA training set were 0.725, 0.695, and 0.697 for 1 year, 2 years, and 3 years, respectively. The AUC values for TCGA validation set were 0.712, 0.688, and 0.715, and 1-, 2-, and 3-years AUC values for all cases were 0.722, 0.692, and 0.703, respectively.

**FIGURE 3:**
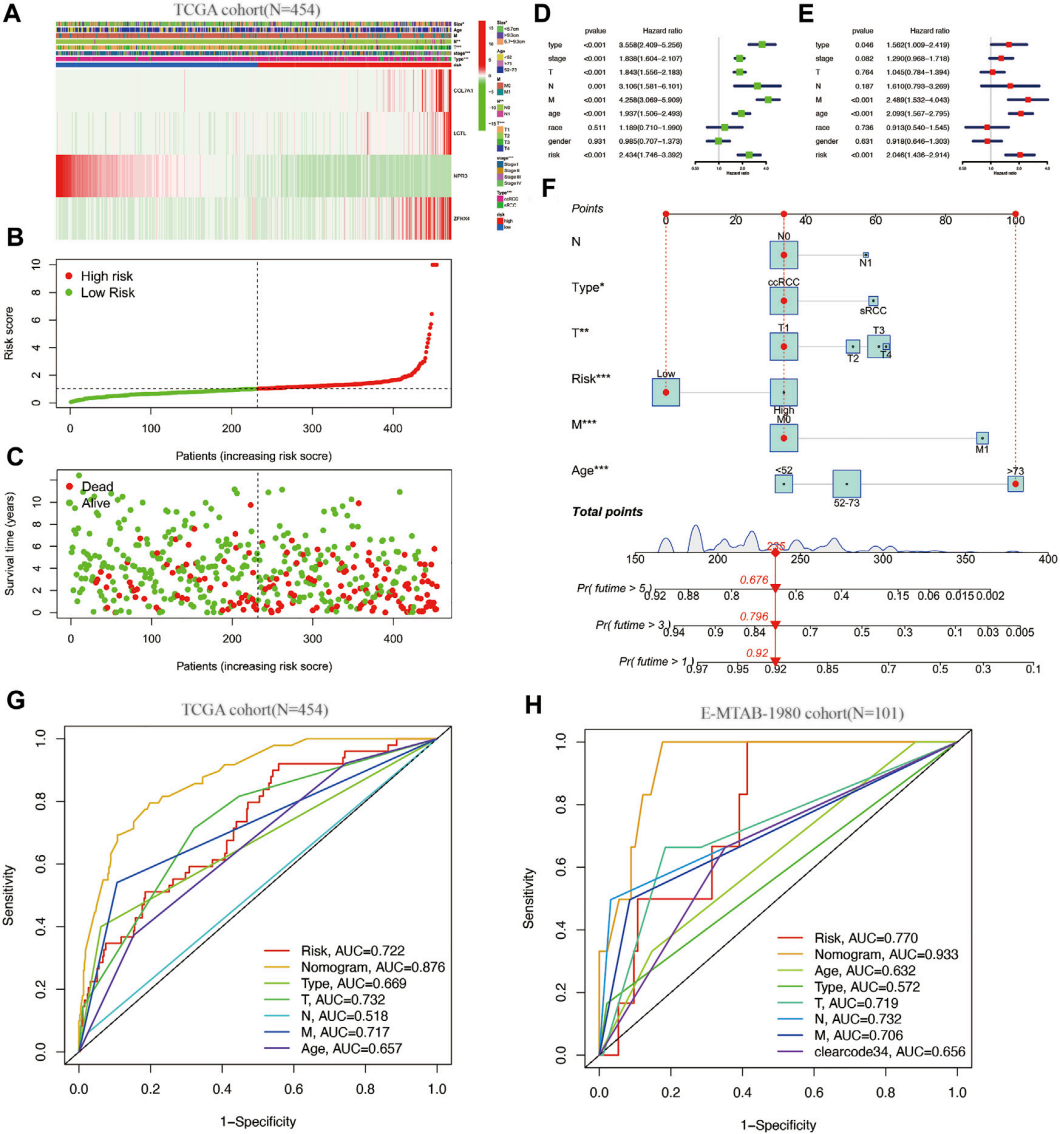
**(A–C)**: clinical information and gene expression heat map of the SAGRI subgroup; **(D)**: one-factor COX regression analysis of SAGRI and clinical information in TCGA cohort; **(E)**: multi-factor COX regression analysis of SAGRI and clinical information in TCGA cohort; **(F)**: nomogram plot constructed for TCGA cohort; **(G)**: ROC curve in TCGA cohort; **(H)**: ROC curve for the external validation cohort.

Heat maps of the expression of these four genes in the 454 cases were plotted, and the relationship of clinical information between the high and low risk groups was compared (Figures 3A–C). The figure clearly shows that three genes, COL7A1, LCTL, and ZFHX4, were highly expressed and NPR3 was downregulated in the high-risk group. Tumor subtype, Stage, T stage, N stage, and tumor size were statistically different between the high and low risk groups (Figure 3A).

The effects of clinical information and the risk model on prognosis were examined using univariate COX regression and multifactorial Cox regression, respectively. The results showed that tumor subtype, Stage, T stage, N stage, M stage, tumor size, age, and SAGRI were influential in prognosis, but only tumor subtype, Stage, M stage, age, and SAGRI were independent risk factors for prognosis in the multifactorial Cox regression (Figures 3D,E). Tumor subtype, Stage, T stage, N stage, M stage, tumor size, age, and SAGRI were included in the construction of the nomogram, which was created by COX regression using the “regplot” and “rms” packages (Figure 3F). The ability of the nomogram (AUC = 0.876) to discriminate was higher than that of TNM stage or other clinical information (Figure 3G). The discriminatory ability of the nomogram (AUC = 0.933) and SAGRI (AUC = 0.770) was verified using the E-MTAB-1980 cohort and compared using clearcode34 (AUC = 0.656) and other clinical information (Figure 3H). The nomogram and risk score model in this study had better discriminatory ability.

### Immune characteristics of different SAGRI subgroups

**FIGURE 4:**
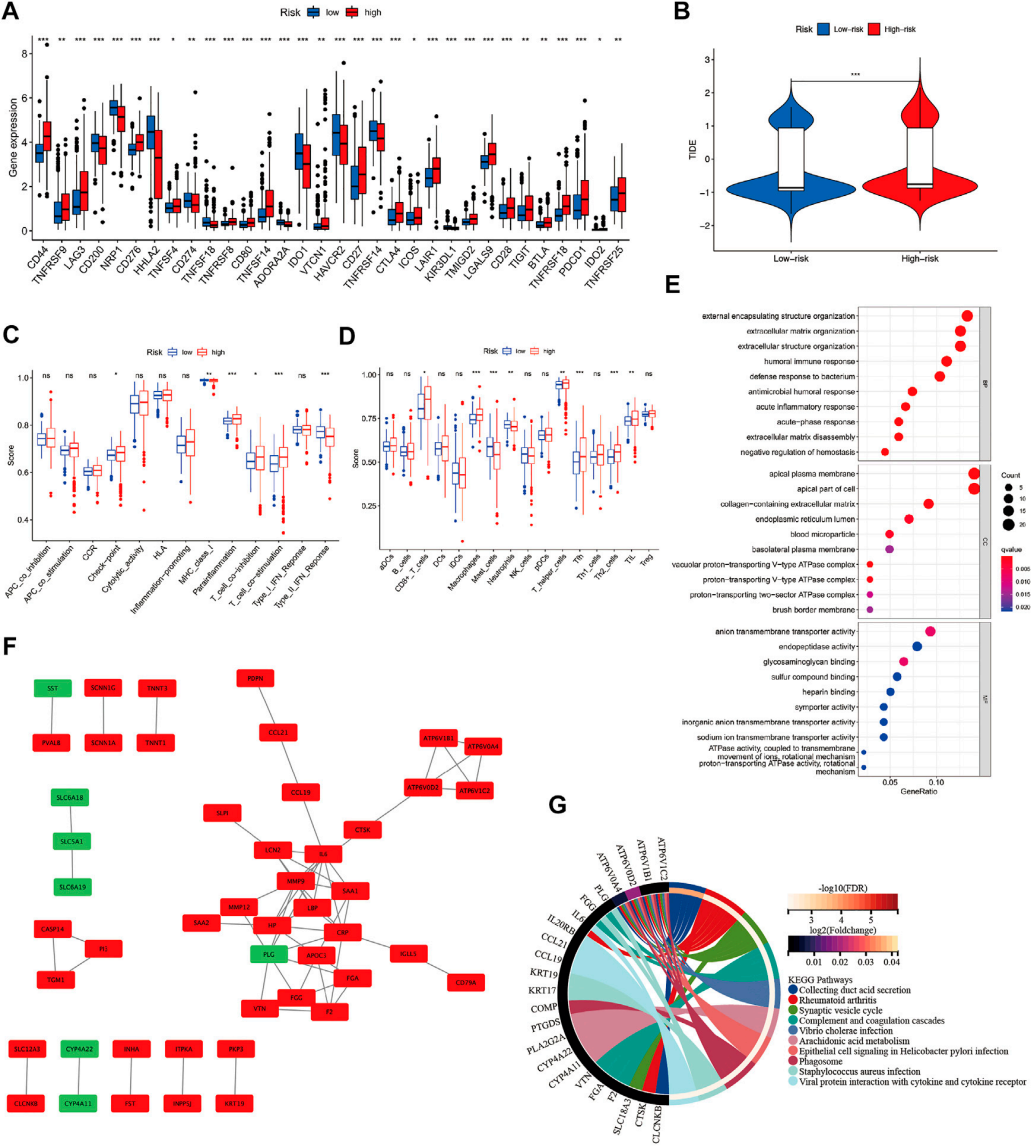
**(A)** Differences in immune checkpoint gene expression between SAGRI subgroups; **(B)** Differences in TIDE scores between SAGRI subgroups; **(C,D)** Differences in immune cells and immune function between SAGRI subgroups; **(E)** Results of GO enrichment analysis between SAGRI subgroups; **(F)** Differential gene-protein interactions between SAGRI subgroups; **(G)** Results of KEGG enrichment analysis between SAGRI subgroups.

The expression of immune checkpoint-related genes was compared between sarcomatoid differentiated renal cell renal carcinoma and clear cell renal carcinoma using a nonparametric test. In this study, 22 immune checkpoint genes were highly expressed in the SAGRI-HIGH subgroup and 10 genes were highly expressed in the SAGRI-LOW subgroup in TCGA cohort (Figure 4A). Notably, PDCD1 was highly expressed in the SAGRI-HIGH subgroup, but the immunotherapy effect of TCGA cohort assessed using the TIDE score suggested that the SAGRI-HIGH subgroup may have had a slightly lower treatment effect than the SAGRI-LOW subgroup (Figure 4B).

Check-point, Parainflammation, T cell co-inhibition, and T cell co-stimulation were higher in the SAGRI-HIGH subgroup than in the SAGRI-LOW subgroup. MHC class I, Type II IFN response was lower than in the SAGRI-LOW subgroup (Figure 4C). The results of the immune cell content analysis are shown in Figure 4D. CD8^+^ T cells, macrophages, T helper cells, Tfh, Th2_cells, and TIL content were higher in the SAGRI-HIGH subgroup than in the SAGRI-LOW subgroup, where the content of mast cells and neutrophils was lower than in the SAGRI-LOW subgroup.

### Identification of hub genes among the SAGRI subgroups

**FIGURE 5:**
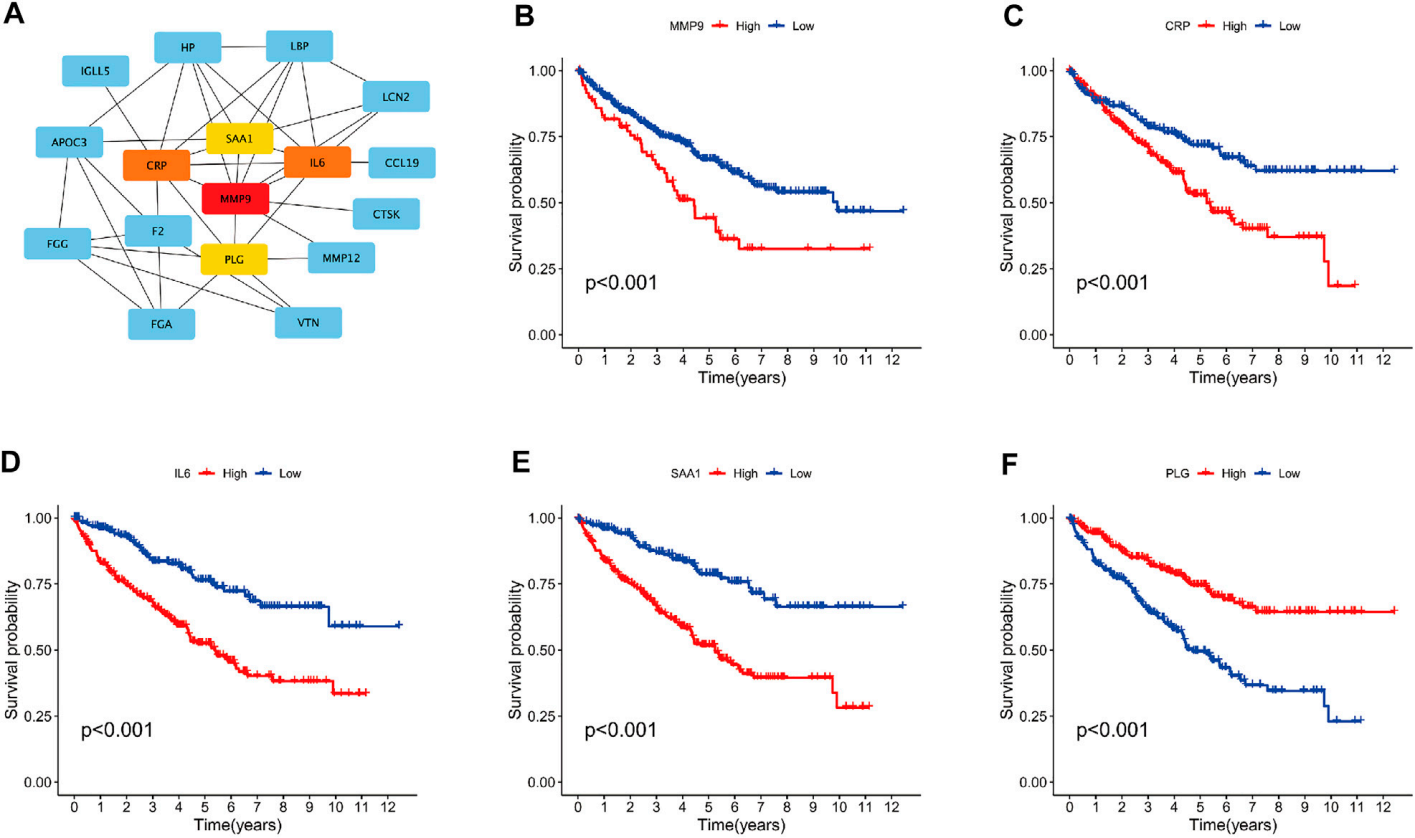
**(A)** Graph of hub gene relationships among SAGRI subgroups; **(B–F)**: survival curve of hub genes in TCGA cohort.

Differential gene analysis was performed between the SAGRI-HIGH and SAGRI-LOW groups using the R package “limma”, and the screening conditions were set as |logFC| ≥ 2 and FDR ≤0.05. The 133 genes were upregulated and 12 genes were downregulated in the SAGRI-HIGH subgroup. The 145 differential mRNAs obtained were subjected to enrichment analysis. Gene Ontology (GO) (Gene Ontology Consortium, 2015) analysis showed that the differential mRNAs were mainly enriched in external encapsulating structure organization and extracellular matrix (Figure 4E). The Kyoto Encyclopedia of Genes and Genomes (KEGG) (Kanehisa and Goto, 2000) showed that the differential mRNAs were mainly enriched in external encapsulating structure organization, extracellular matrix organization, apical plasma membrane, apical part of cell, and anion transmembrane transporter activity. Enrichment analysis showed that differentially expressed mRNAs were mainly enriched in the PPAR signaling pathway, IL-17 signaling pathway, NF-kappa B signaling pathway, Arachidonic acid metabolism, and the TNF signaling pathway (Figure 4G). Protein interactions between differential genes were analyzed using the STRING online website, and results are shown in Figure 4F. In the figure, red represents upregulated genes in the SAGRI-HIGH subgroup and green represents downregulated genes. Key nodes in the protein interaction network were identified using Cytoscape software, and the most central gene was found to be MMP9, followed by CRP, IL6, SAA1, and PLG (Figure 5A). The K-M survival curves of the five hub genes in TCGA cohort were plotted using R language (Figures 5B–F). We found that both high and low expression of the five hub genes were closely associated with the prognosis of clear cell renal cell carcinoma.

## Discussion

Sarcomatoid renal cell carcinoma (sRCC) is one of the most aggressive types of kidney cancer (Przybycin et al., 2014; Kyriakopoulos et al., 2015). Therefore, we screened for genes related to sarcomatoid differentiation that may be used to assess prognosis in renal cell carcinoma. In this study, we used propensity score matching to strictly match sarcomatoid renal cell carcinoma cases with clear cell renal cell carcinoma cases in TCGA database. All sarcomatoid differentiation was found to be from clear cell carcinoma, and the differentially expressed genes in sarcomatoid differentiation screened on this basis were more credible. We screened a total of 393 upregulated mRNAs and 46 downregulated mRNAs in sarcomatoid renal cell carcinoma.

In this study, we constructed a prognostic stratification model for kidney cancer using the screened sarcomatoid differentiation-related genes. The constructed model was found to be more accurate in predicting longer-term prognosis. The closeness of the validation set results to the training set indicated that no overfitting occurred. Recent studies suggest that NPR3 may be negatively associated with adenylyl cyclase and the MAP kinase signaling pathway (mitogen-activated protein kinase, MAPK) (Prins et al., 1996; Lelièvre et al., 2001), and the adenylate cyclase can stimulate the MAPK signaling pathway (Breckler et al., 2011; Tang et al., 2013). Several studies have also shown that the MAPK signaling pathway plays an activating role in the development, metastasis, and angiogenesis of several human malignancies, including renal cell carcinoma (Webb et al., 1998; Ward et al., 2001; Huang et al., 2008). COL7A1 encodes the alpha chain of type VII collagen. Studies focused on COL7A1 demonstrated that COL7A1 is significantly upregulated in gastric cancer tissues and is an independent risk factor for poor prognosis in gastric cancer (Webb et al., 1998).

We further performed a pan-cancer survival analysis of TCGA database (Supplementary Tables S4–7) for the four genes modeled. COL7A1 suggested poor prognosis in a variety of TCGA tumors: kidney cancer (KIRC: HR = 1.36; KIRP: HR = 1.23; KICH: HR = 1.45), mesothelioma (MESO: HR = 1.36), adrenocortical carcinoma (ACC: HR = 1.37), lung adenocarcinoma (LUAD: HR = 1.11), pancreatic adenocarcinoma (PAAD: HR = 1.17), and cholangiocarcinoma (CHOL: HR = 1.40) (Supplementary Figure S2). LCTL in glioma (GBMLGG: HR = 1.73; LGG: HR = 1.60), kidney cancer (KIRC: HR = 1.31; KICH: HR = 2.92; KIRP: HR = 1.45) ZFH, mesothelioma (MESO: HR = 1.28), uveal melanoma (UVM: HR = 1.56), bladder urothelial carcinoma (BLCA: HR = 1.15), lung adenocarcinoma (LUAD: HR = 1.14) (Supplementary Figure S3) and ZFHX4 in renal cancer (KIRC: HR = 1.18; KIRP: HR = 1.23), stomach adenocarcinoma (STAD: HR = 1.16), testicular germ cell tumors (THCA: HR = 1.66), bladder urothelial carcinoma (BLCA: HR = 1.09), and uterine corpus endometrial carcinoma (UCEC: HR = 1.15) also suggested poor prognosis (Supplementary Figure S4), but not in uterine carcinosarcoma (UCS: HR = 0.79), glioma (GBMLGG: HR = 0.86) where an association with good prognosis was suggested. NPR3 (Supplementary Figure S5) was found to be a favorable prognostic factor in this study and was differently associated with prognosis in different subtypes of renal cell carcinoma. nPR3 was suggested to be favorable to prognosis in KIRC (HR = 0.80) and KIRP (HR = 0.84), but negative in KICH (HR = 1.87). In addition, NPR3 was also suggested as favorable to prognosis in adrenocortical carcinoma (ACC: HR = 0.83) but not in stomach adenocarcinoma (STAD: HR = 1.15), bladder urothelial carcinoma (BLCA: HR = 1.11), or breast invasive carcinoma (BRCA: HR = 1.10) where it suggested poor prognosis.

The 1-year AUC values for TCGA training cohort of the SAGRI constructed using the four sarcoma-like differentiation-related genes (COL7A1, LCTL, NPR3, ZFHX4) were 0.725 in the training set, 0.712 in the internal validation set, and 0.770 in the external validation set. This indicated that the LASSO regression used in this study did not overfit and the model was reliable. Moreover, the heat map showed that sarcomatoid renal carcinoma was mainly concentrated in the high-risk group as predicted by the model, which also demonstrated better predictive ability of the model for prognosis. To further optimize the prognostic model for renal cell carcinoma, we included the four-gene prognostic model and other clinical information into the multifactorial prognostic analysis and found that tumor subtype (i.e., whether the cancer was sarcomatoid renal carcinoma), stage, whether it was metastatic or not, age, and the four-gene prognostic model were all independent risk factors for renal cell carcinoma. A nomenclature was constructed, and the obtained nomenclature had a 1-year AUC value of 0.875, with a significant improvement in predictive power. The 1-year AUC value was 0.933 in the independent cohort validation and was significantly higher than the Clearcode34 classification. The existing, more mature clinical models for prognostic analysis and prediction of patients with renal cell carcinoma mainly include tools such as TNM staging, UISS score, RCClnc4, and Clearcode34. TNM staging mainly relies on clinical information and pathological information without biological features and cannot effectively distinguish inert tumors from invasive tumors. UISS score is mainly used for limited renal cancer and cannot effectively assess the prognosis of patients with all types of renal cell carcinoma. Moreover, the TNM staging and UISS scoring systems were constructed based on clinical variables with postoperative TNM staging and tumor grade as the main predictors, and these systems lack tumor molecular markers related to kidney cancer prognosis. In contrast, RCClnc4 and Clearcode34 were constructed using genes as the main predictors, based on genetic variables, and lack relevant clinical factors. In the prognosis assessment of renal cell carcinoma patients, both biological and clinical factors are very important.

The above studies failed to effectively combine genetic and clinical factors to establish a model for predicting survival of renal cell carcinoma patients, which is insufficient to assess the prognosis of renal cell carcinoma patients comprehensively and effectively. Owing to the vastly different clinical outcomes of patients with clear cell renal cell carcinoma, especially those with combined sarcomatoid differentiation, this disease requires new prognostic models. Therefore, this study combined biological and clinical factors to construct a comprehensive model for predicting patients with clear cell renal cell carcinoma. Comparing the results of the nomogram constructed in this study with TNM staging and Stage staging, we found that the nomogram was superior to TNM staging and Stage staging as well as Clearcode34 in terms of predictive accuracy and clinical utility.

The discovery of immune checkpoints was an important breakthrough in cancer immunology. In almost all human cancer cells, there is disruption of DNA integrity due to insertions, deletions, substitutions of nucleotides, chromosomal deletions, duplications, or translocation events. This genetic heterogeneity provides the basis for clonal evolution, allowing cancer cells to become progressively resistant to therapy. However, this also generates neoantigens that the autoimmune system can recognize as foreign substances (Ribas and Wolchok, 2018). However, multiple mechanisms can suppress the antitumor immune response, making it impossible to kill tumor cells even when tumor-killing T cells infiltrate the tumor microenvironment. Among them, immune checkpoints are immune molecules present in the immune system that are responsible for upregulating (co-stimulatory molecules) or downregulating immune system signals. In TCGA cohort, 22 immune checkpoint genes were highly expressed in the SAGRI-HIGH subgroup and 10 genes were highly expressed in the SAGRI-LOW subgroup (Figure 4A). Notably, PDCD1 was highly expressed in the SAGRI-HIGH subgroup, but the immunotherapy effect of TCGA cohort assessed using the TIDE score suggested that the SAGRI-HIGH subgroup may have had a slightly worse treatment effect than the SAGRI-LOW subgroup (Figure 4B). This may be due to the higher malignancy of the SAGRI-HIGH subgroup itself or other confounding factors; however, the use of immunotherapy in the SAGRI-HIGH subgroup may still be an option worth considering. In particular, the expression of CTLA-4 and PDCD1 was higher in the SAGRI-HIGH subgroup, also suggesting that the SAGRI-HIGH subgroup may be more sensitive to immune checkpoint therapy. In T-cell activation, CTLA-4 and PD-L1/PD-1 are crucial immune co-suppressive signals, and immune checkpoint inhibitors targeting them have been widely used in recent years for a variety of tumors.

For the first-line treatment of metastatic kidney cancer, the CheckMate 214 study found that the combination of the PD1 antibody nabumab and the CTLA-4 antibody epitumumab was significantly superior to the targeted drug sunitinib in terms of overall survival for intermediate- and high-risk metastatic kidney cancer in International Metastatic Renal-Cell Carcinoma Database Consortium (IMDC) (Motzer et al., 2018). The KEYNOTE-426 study showed that the pablizumab combined with axitinib treatment group had significantly better progression-free survival than the sunitinib-treated group in metastatic kidney cancer (Rini et al., 2019). The results of these two studies have brought the treatment of metastatic kidney cancer from the era of targeted drugs to a new era of immunotherapy. Currently, major guidelines such as NCCN and EAU recommend nabumab in combination with epirubicin or pablizumab in combination with axitinib as first-line treatment options for intermediate- and high-risk metastatic kidney cancer in IMDC (Jonasch, 2019). Recent results from two other phase III clinical trials (JAVELIN Renal 101 and Immotion 151) also showed that in PD-L1-positive patients with metastatic kidney cancer, treatment with the PD-L1 antibody avelumab in combination with axitinib had longer progression-free survival than the sunitinib treated group (Motzer et al., 2019), while patients treated with the PD-L1 antibody atezolizumab and VEGF antibody bevacizumab had longer progression-free survival than the sunitinib group (Motzer et al., 2022). A retrospective analysis suggested that the combination of immune checkpoint inhibitors for metastatic sarcomatoid kidney cancer is superior to conventional, targeted agents alone (Hanif et al., 2019).

We analyzed the differentially expressed genes between the SAGRI-HIGH and SAGRI-LOW groups using the STRING website and found five key hub genes, the most core gene being MMP9, followed by CRP, IL6, SAA1, and PLG. All these genes are closely related to the prognosis of ccRCC, and studies targeting these genes may be important to improving prognosis and outcomes in ccRCC patients.

There were some limitations to this study. First, this was a retrospective study, and although propensity score matching has been used to pairwise screen for sarcomatoid differentiation trait factors, selection bias is difficult to avoid in the overall context. Therefore, follow-up multicentre prospective studies are needed to further validate the accuracy of the nomogram prognostic model. Second, sarcomatoid differentiated renal cell carcinoma tumors are inherently heterogeneous, which makes it difficult to ensure the quality of TCGA database sampling. In addition, the proportion of sarcomatoid differentiation was not included in the analysis, introducing potential bias in the analysis results. Third, although this study found that four genes (COL7A1, LCTL, NPR3, and ZFHX4), had a large impact on the prognosis of renal cell carcinoma and analyzed their relationship with survival in multiple cancers, the specific molecular biology and cell biology mechanisms need to be further investigated.

## Conclusion

In this study, we identified genes related to sarcoma-like differentiation which were then used to construct a SAGRI model of renal clear cell carcinoma. We then combined the genes with clinical information to construct a nomogram. The SAGRI model and nomogram in this study had high specificity and sensitivity in both TCGA training and validation sets and were validated in an independent cohort. This study also analyzed the genetic differences and immune differences among SAGRI subtypes. Targeted therapy against immune checkpoints may be key to the treatment of renal cell carcinomas with poor prognosis.

## Data Availability

The original contributions presented in the study are included in the article/Supplementary Material further inquiries can be directed to the corresponding author.
